# Human anti-CD30 recombinant antibodies by guided phage antibody selection using cell panning

**DOI:** 10.1054/bjoc.2000.1226

**Published:** 2000-06-15

**Authors:** A Klimka, B Matthey, R C Roovers, S Barth, J-W Arends, A Engert, H R Hoogenboom

**Affiliations:** Laboratory of Immunotherapy, Department of Internal Medicine I, University Hospital Cologne, Joseph Stelzmann Str. 9, Cologne, 50931, Germany; Department of Pathology, Maastricht University, PO Box 5616, Maastricht, MD, 6200, the Netherlands

**Keywords:** CD30, phage display, chain shuffling, human antibody, guided selection

## Abstract

In various clinical studies, Hodgkin’s patients have been treated with anti-CD30 immunotherapeutic agents and have shown promising responses. One of the problems that appeared from these studies is the development of an immune response against the non-human therapeutics, which limits repeated administration and reduces efficacy. We have set out to make a recombinant, human anti-CD30 single-chain variable fragment (scFv) antibody, which may serve as a targeting moiety with reduced immunogenicity and more rapid tumour penetration in similar clinical applications. Rather than selecting a naive phage antibody library on recombinant CD30 antigen, we used guided selection of a murine antibody in combination with panning on the CD30-positive cell line L540. The murine monoclonal antibody Ki-4 was chosen as starting antibody, because it inhibits the shedding of the extracellular part of the CD30 antigen. This makes the antibody better suited for CD30-targeting than most other anti-CD30 antibodies. We have previously isolated the murine Ki-4 scFv by selecting a mini-library of hybridoma-derived phage scFv-antibodies via panning on L540 cells. Here, we report that phage display technology was successfully used to obtain a human Ki-4 scFv version by guided selection. The murine variable heavy (VH) and light (VL) chain genes of the Ki-4 scFv were sequentially replaced by human V gene repertoires, while retaining only the major determinant for epitope-specificity: the heavy-chain complementarity determining region 3 (CDR3) of murine Ki-4. After two rounds of chain shuffling and selection by panning on L540 cells, a fully human anti-CD30 scFv was selected. It competes with the parental monoclonal antibody Ki-4 for binding to CD30, inhibits the shedding of the extracellular part of the CD30 receptor from L540 cells and is thus a promising candidate for the generation of anti-CD30 immunotherapeutics. © 2000 Cancer Research Campaign


*British Journal of Cancer* (2000) **83**(2), 252–260
© 2000 Cancer Research Campaign
doi: 10.1054/ bjoc.2000.1226, available online at http://www.idealibrary.com on IDEAL®

### Human anti-CD30 recombinant antibodies by guided phage antibody selection using cell panning

**A Klimka[1,2], B Matthey[1], RC Roovers[2], S Barth[1], J-W Arends[2], A Engert [1] and HR Hoogenboom[2]**

[1]Laboratory of Immunotherapy, Department of Internal Medicine I, University Hospital Cologne, Joseph Stelzmann Str. 9, 50931 Cologne, Germany;
[2]Department of Pathology, Maastricht University, PO Box 5616, 6200 MD Maastricht, the Netherlands

**Summary** In various clinical studies, Hodgkin's patients have been treated with anti-CD30 immunotherapeutic agents and have shown promising responses. One of the problems that appeared from these studies is the development of an immune response against the non-human therapeutics, which limits repeated administration and reduces efficacy. We have set out to make a recombinant, human anti-CD30 single-chain variable fragment (scFv) antibody, which may serve as a targeting moiety with reduced immunogenicity and more rapid tumour penetration in similar clinical applications. Rather than selecting a naive phage antibody library on recombinant CD30 antigen, we used guided selection of a murine antibody in combination with panning on the CD30-positive cell line L540. The murine monoclonal antibody Ki-4 was chosen as starting antibody, because it inhibits the shedding of the extracellular part of the CD30 antigen. This makes the antibody better suited for CD30-targeting than most other anti-CD30 antibodies. We have previously isolated the murine Ki-4 scFv by selecting a mini-library of hybridoma-derived phage scFv-antibodies via panning on L540 cells. Here, we report that phage display technology was successfully used to obtain a human Ki-4 scFv version by guided selection. The murine variable heavy (VH) and light (VL) chain genes of the Ki-4 scFv were sequentially replaced by human V gene repertoires, while retaining only the major determinant for epitope-specificity: the heavy-chain complementarity determining region 3 (CDR3) of murine Ki-4. After two rounds of chain shuffling and selection by panning on L540 cells, a fully human anti-CD30 scFv was selected. It competes with the parental monoclonal antibody Ki-4 for binding to CD30, inhibits the shedding of the extracellular part of the CD30 receptor from L540 cells and is thus a promising candidate for the generation of anti-CD30 immunotherapeutics. © 2000 Cancer Research Campaign

**Keywords**: CD30; phage display; chain shuffling; human antibody; guided selection

Although monoclonal antibodies (moab) raised by hybridoma technology (Köhler and Milstein, 1975) have been demonstrated to be very useful in research and diagnosis, they are somewhat problematic as binding moieties in immunotherapeutic agents for the treatment of tumours. Apart from their relatively large size (150 kDa), which makes it difficult to penetrate into solid tumours, these non-human antibodies generate an immune response resulting in serious side-effects such as serum sickness or anaphylactic shock, which prevent long-term treatment of cancer patients (Shawler et al, 1985). It is also documented that this human anti-mouse antibody (HAMA) response causes a rapid blood clearance of these reagents, which diminishes their efficacy (Khazaeli et al, 1994).

To circumvent these problems, two strategies have been followed. First, a reduction of the molecular size of the binding moiety using Fab fragments or even just the variable fragments of an antibody as a single-chain variable fragment (scFv) has significantly reduced the target surface for an immune response and thus the immunogenicity. Secondly, the use of humanized proteins like chimaeric or CDR-grafted, or even fully human antibodies or antibody fragments, has been demonstrated to reduce their immunogenicity (Meredith et al, 1993).

*Received 6 December 1999*
*Revised 6 March 2000*
*Accepted 10 March 2000*

*Correspondence to:* HR Hoogenboom

Because of technical problems and difficulties in retrieving suitable human donors, it is complicated to raise human hybridomas by conventional techniques. However, the progress in molecular biology has offered different ways to evade this restriction. One possibility is the use of transgenic mice carrying human immunoglobulin genes. These mice can be used to generate hybridomas secreting human antibodies (Brüggemann and Neuberger, 1996). Another way is the use of human V-gene libraries expressed and displayed on phage and selection of antigen-specific antibodies therefrom (Hoogenboom, 1997; Winter et al, 1994). These libraries can be derived from immunized or non-immunized donors or even generated synthetically (Hoogenboom et al, 1998). Indeed, from very large phage libraries, high-affinity antibodies to many different target antigens can be selected (Hoogenboom, 1997). This *in vitro* selection procedure is subjected to a series of biases introduced by library preparation, selection conditions and the screening protocol. Strong biases in selected populations can arise, in particular when selecting on complex antigenic targets (Hoogenboom et al, 1999; Persic et al, 1999).

Therefore, it sometimes remains difficult to retrieve antibodies with desired properties like recognition of a unique epitope, induction of a post-binding signal transduction or internalization upon binding to a cell-surface receptor on the target cell (McCall et al, 1998). Hybridoma-derived antibodies with such characteristics are sometimes available, and may be converted to human versions using a method termed 'guided selection' (Jespers et al, 1994): by two consecutive chain-shuffling procedures, the rodent antibody domains are swapped for human domains, using phage display technology (library construction and selection on antigen) to retrieve the best-matching partner. Our goal was to obtain a fully human antibody from the well-characterized murine moab Ki-4, which recognizes the CD30 receptor.

CD30 was originally identified by Schwab et al (1982) as the antigen abundantly expressed on Hodgkin–Reed Sternberg cells (H–RS) in primary Hodgkin's lymphoma and recognized by the first anti-CD30 moab Ki-1. Expression of CD30 in high copy numbers on the cell surface has also been reported for a subset of non-Hodgkin lymphomas (NHL), virally transformed B- and T-cell lines, a subform of large-cell anaplastic lymphoma ($CD30^+$-LCAL), embryonal carcinomas, malignant melanomas and mesenchymal tumours (Gruss and Dower, 1995). The CD30 receptor is therefore a useful clinical and pathological tumour marker for these diseases and a good target for immunotherapy.

Here we report the synthesis of a human anti-CD30 scFv (hAK30) on the basis of the murine anti-CD30 moab Ki-4. Murine moab Ki-4, which shows no detectable cross-reactivity with vital human organs, has successfully been used as part of a chemically linked ricin A immunotoxin in vivo (Schnell et al, 1995) and also as a scFv in a *Pseudomonas* exotoxin A-based recombinant immunotoxin in vitro (Klimka et al, 1999). Therefore, we exchanged the murine variable heavy (VH) and light (VL) chain genes with human counterparts with respective selections on the CD30-positive Hodgkin cell line L540. This strategy allowed the construction of a fully human anti-CD30 scFv (hAK30) with the same binding specificity as moab Ki-4, and in which only the VH(CDR3) and framework 4 sequences are derived from the parental antibody. This scFv may serve as a useful building block for the synthesis and engineering of different fusion proteins, such as scFv coupled to toxins, enzymes, or, in connection with other targeting molecules, as bispecific agents (Huston et al, 1993). It is a promising candidate to use as immunotherapeutic agent for the treatment of CD30-positive malignancies.

### MATERIAL AND METHODS

### Cell lines

The Hodgkin-derived cell line L540 (Diehl et al, 1981) and the hybridoma cell lines BW702 (Bosslet et al, 1989), Ki-3, Ki-4, Ki-6, Ki-7 (Horn-Lohrens et al, 1995) and BerH2 (Schwarting et al, 1989) were maintained in RPMI 1640 medium (GIBCO-BRL, Rockville, MD, USA) supplemented with 10% (v/v) FCS, 100 µg $ml^{-1}$ streptomycin, 200 units $ml^{-1}$ penicillin and 2 mM L-glutamine (10% FCS-medium). All cells were cultured at 37°C in a 5% $CO_2$ humidified atmosphere.

### Bacterial strains and plasmids

*E.coli* XL1-Blue (supE44, hsdR17, recA1, endA1, gyr, A46, thi, relA1, lacF′. $proA^+$ $B^+$ $lacI^q$, lacZ_M15, Tn10($tet^r$)] were obtained from Stratagene (La Jolla, CA, USA). *E.coli* TG1 (K12_(lac-pro), supE, thi, hsdD5/F′traD36, $proA^+$ $B^+$ , $lacI^q$, lacZ_M15) and *E.coli* HB2151 (K12_(lac-pro), ara, $nal^r$, thi/F′, $proA^+$ $B^+$, $lacI^q$, lacZ_M15) were purchased from Pharmacia (Uppsala, Sweden). The phagemid vector pCANTAB6 (McCafferty et al, 1994) is used for N-terminal fusion of scFv fragments to the minor coat protein p3 of filamentous phage M13 using Sfi I (Nco I)/Not I restriction sites. An amber-stop codon between the scFv-gene and the bacteriophage gene 3 allows the expression of soluble fragment or phage-displayed scFv, in an *E.coli* non-suppressor or suppressor strain, respectively.

### Chain shuffling of murine Ki-4 V-genes

The murine Ki-4 scFv was synthesized as described (Klimka et al, 1999). From this scFv, the CDR3-linker-VL-gene fragment was amplified by polymerase chain reaction (PCR) using 30 cycles of 94°C for 1 min, 55°C for 1 min and 72°C for 2 min, with the primers VH-FR3-BACK (5′-GAC ACG GCY GTR TAT TAC TGT-3′) and FD-TET-SEQ (5′-TTT GTC GTC TTT CCA GAC GTT AGT-3′) and the proof-reading *Pfu*-polymerase (Stratagene, La Jolla, CA, USA) according to the manufacturer's instructions. Simultaneously, human VH genes lacking the CDR3-FR4 sequence were amplified from the pHEN1-human scFv repertoire made by Marks et al (1991), using the primers pUC-REV (5′-CAG GAA ACA GCT ATG AC-3′) and VH-FR3-FOR (5′-ACA GTA ATA YAC RGC CGT GTC-3′). For PCR assembly of the amplified fragments, 250 ng of each were combined in a 50 µl mixture and cycled seven times (94°C for 1.5 min, 65°C for 1 min and 72°C for 2 min) to join the fragments. The reaction mixture was then amplified for 30 cycles (94°C for 1 min, 55°C for 2 min and 72°C for 2 min) after the addition of the outer PCR primers pUC-REV/FD-TET-SEQ. Assembly products were digested with Sfi I/Not I and ligated into the phagemid vector pCANTAB6. The ligation mix was purified by phenol extraction and ethanol precipitation and dissolved in 20 µl $H_2O$. The DNA solution was transfected into 100 µl *E.coli* TG1 by electroporation as described elsewhere (Dower et al, 1988). The cells were grown for 1 h in 2×TY medium at 37°C before plating on 2×TY agar medium containing 100 µg ampicillin $ml^{-1}$ and 2% (w/v) glucose (2×TY-Amp-Glu).

Five different selected human VH genes, determined by DNA-fingerprint analysis as described elsewhere (Marks et al, 1991) from 20 CD30-reactive half-human scFvs, were amplified with primers pUC-REV/VH1-FOR-Xho (5′-CCG CCT CCA CCA CTC GAG ACG GTG ACC GTG GTC CC-3′) using Pfu-polymerase, ligated into pCANTAB6 using restriction enzymes Sfi I/Xho I and electroporated into *E.coli* XL1-Blue. After sequencing of the human VH genes, they were cloned into the pHEN1-VL repertoire (Marks et al, 1991) using the restriction sites Sfi I/Xho I and transfected into *E.coli* TG1 by electroporation as described above. After selection, the human anti-CD30 scFv (hAK30) was finally cloned into pCANTAB6 using the restriction enzymes SfiI/NotI for expression as His-tagged protein.

### Selection of phage on the Hodgkin-derived cell line L540

The resulting repertoires of transformed bacteria containing the murine Ki-4 VL linked to human VH repertoire in phagemid vector pCANTAB6 or selected human VH-genes linked to the human VL repertoire in pHEN1, were rescued with helper phage M13K07 as described (Marks et al, 1991). The selection procedure is described elsewhere (Klimka et al, 1999). Briefly, $5 \times 10^5$ L540 cells were incubated with 1 ml of $1 \times 10^{13}$ cfu $ml^{-1}$ phage in 2% (w/v) MPBS (2% Marvel skimmed milk powder in PBS) for 1 h at room temperature (RT). After washing the cells ten times with

© 2000 Cancer Research Campaign

5 ml 2% MPBS and two times with 5 ml PBS by spinning (300 ***g***, 3 min, RT) and resuspending respectively, binding phage were eluted with 50 mM HCl and remaining cell debris was spun down (300 ***g***, 5 min, RT) after neutralization with 1 M Tris-Cl, pH 7.4. Phage-containing supernatant (SN) was mixed with 3 ml 2×TY-Glu medium and used to transfect logarithmically growing *E.coli* TG1 cells for 30 min at 37°C before plating on 2×TY-Amp-Glu agar medium.

### FACS analysis

Cell binding of phage-displayed scFvs was demonstrated by FACS analysis. $5 \times 10^5$ L540 target cells were washed in PBS containing 2% (w/v) skimmed milk powder and 0.05% (w/v) sodium azide (2% MPBS/$N_3$–) and then incubated for 1 h at 4°C with the respective phage or moabs Ki-3 or Ki-4 in 2% MPBS/$N_3$– respectively. Bound phage were detected with a sheep anti-fd serum (Pharmacia, Uppsala, Sweden; 0.02% (v/v) in 2% MPBS/$N_3$–) and FITC-labelled rabbit-anti-sheep IgG (Dianova, Hamburg, Germany; 2% (v/v) in MPBS/$N_3$–). Bound monoclonal antibodies were detected with FITC-conjugated goat-anti-mouse IgG (Becton & Dickinson, Heidelberg, Germany); cells were analysed on a FACScan (Becton & Dickinson). For competition FACS analysis, approximately $10^{12}$ cfu of phage displaying scFv were mixed with 50 μl of unpurified supernatant from hybridomas secreting moab Ki-3 or moab Ki-4, respectively, resulting in a phage vs moab ratio of approximately 1/1. The mixtures were incubated with the target cells and bound phage were subsequently detected as described.

### Sequencing

The scFv-genes were sequenced by the dideoxy chain termination method (Sanger et al, 1977) using Dye-Terminator mix (Perkin Elmer, Norwalk, CO, USA) and the oligonucleotides FD-TET-SEQ and pUC-REV. Products of the sequencing reaction were analysed on a semi-automated ABI Prism sequencer (Perkin Elmer). The nucleic acid sequences of the V regions were compared to the Kabat database of V genes (Kabat and Wu, 1991) and Sanger Centre database (http://www.sanger.ac.uk) to determine the V-gene family and germline V-gene segments.

### Purification of recombinant, human, soluble CD30-His

Cloning of the extracellular part of human CD30 receptor fused to a $His_6$-tag into the eukaryotic expression vector pcDNA3 (Invitrogen, Groningen, The Netherlands) is described elsewhere (Barth et al, 2000). 250 ml supernatant of COS-1 cells transfected with sCD30-His-pcDNA3 plasmid was collected, filtered and incubated with 2 ml Talon™ resin (Clontech, Heidelberg, Germany) for 2 h at 4°C for IMAC purification. The resin was subsequently washed with Tris-buffer (20 mM Trisbase, 100 mM NaCl, pH 8.0) and Tris-buffer, 5 mM Imidazol until the $OD_{280\ nm}$ dropped to 0.001. The sCD30-His protein was then cluted with 250 mM Imidazol in Tris-buffer and dialysed against PBS ovn at 4°C.

### Purification of scFv

*E.coli* HB2151, harbouring the respective scFv genes in pCANTAB6 were used to inoculate 750 ml of 2×TY medium containing 100 μg ml$^{-1}$ ampicillin and 0.1% (w/v) glucose. The culture was grown at 37°C to an $OD_{600\ nm}$ of 0.9 and then supplemented with 1 mM isopropylthio-β-D-galactoside (IPTG) for induction of soluble scFv expression. After 4 h of induction at 30°C, the cells were pelleted and resuspended in 8 ml ice-cold TES (200 mMTris-HCl; 0.5 mM EDTA; 500 mM sucrose). After incubation for 5 min on ice, 8.8 ml of TES/$H_2O$ (1:3) were added and the bacterial suspension was incubated on ice for an additional 20 min. Bacteria were pelleted, SN was collected and the pellet was resuspended in 10 ml TES/15 mM $MgSO_4$ and incubated on ice for 15 min. After centrifugation for 5 min at 300 ***g*** the supernatants were mixed and centrifuged at 13 000 ***g*** for 10 min to remove cell debris. The resulting periplasmic fraction was dialysed against Tris-buffer (20 mM Trisbase, 100 mM NaCl, pH 8.0) ovn at 4°C and the scFvs were purified by IMAC using Talon™ resin (Clontech, Palo Alto, USA) as described for the sCD30-His protein.

The human scFv hAK30 was additionally expressed under high-salt stress induction as described elsewhere (Barth et al, 2000). In brief, 2 L bacterial culture were grown at 28°C in TB-medium containing 0.5 mM $ZnCl_2$ and 0.1 M potassium phosphate buffer, pH 7.5 till $OD_{600nm}$ of 1.6. The culture was supplemented with 0.5 M sorbitol, 0.7 M NaCl, 10 mM betain and after 15 min expression was induced by addition of 1 mM IPTG. After overnight growth, bacteria were centrifuged and the pellet was snap-frozen in liquid nitrogen and resuspended in 75 mM Tris-buffer, pH 8.0 containing 10% glycerol, 300 mM NaCl, 2 mM EDTA, 5 mM DTT and Complete™ protease inhibitor (Boehringer Mannheim, Mannheim, Germany). Proteins were extracted by sonification and centrifugation, desalted by gelchromatography using a desalting column (Pharmacia, Uppsala, Sweden) and scFv was isolated by IMAC using Ni-NTA resin (Qiagen, Hilden, Germany).

Eluted protein was thoroughly dialysed against PBS and visualized by gelfiltration, SDS-PAGE and immunoblotting. The final concentration was determined from a scanned Coomassie-stained SDS-PAGE with BSA-dilutions as standards and performing densitometrical analysis with Multi-Analyst software (Bio-Rad, Munich, Germany).

### Determination of relative binding affinities of anti-CD30 antibodies

To determine the relative binding affinities of the anti-CD30 antibodies, purified recombinant sCD30-His (70 nM) was incubated for 1 h at RT in duplicates, with dilution series of the respective purified scFvs, the Ki-4 Fab fragment prepared as described elsewhere (Smith, 1993), or the moab Ki-4, respectively. Unbound sCD30-His antigen was detected in a CD30 (Ki-1 antigen)-ELISA kit (DAKO, Glostrup, Denmark) where the coated anti-CD30 antibody BerH2 binds to the same CD30-epitope as the investigated antibodies. sCD30-His captured by BerH2 was detected by peroxidase-conjugated anti-CD30 antibody Ki-1, which binds to a different epitope. The ELISA was performed according to the manufacturer's instructions and extinction at 450 nm was measured. The antibody concentration at which the $OD_{450}$ dropped to 50% of maximum extinction was taken as the apparent $K_d$.

### Measurement of shed sCD30 receptor

$2 \times 10^5$ L540 cells were washed three times with 10 ml fresh 10% FCS medium and incubated with 1/10 diluted supernatants of

 © 2000 Cancer Research Campaign

**Table 1** Selection of half-human (**A**) and human (**B**) anti-CD30 phage antibodies on Hodgkin cell line L540.

| Phage clones | Input titre (cfu) | Output titre (cfu) | Ratio (output/input) | Frequency of positive clones in whole-cell ELISA[a] |
|---|---|---|---|---|
| **A** | | | | |
| Before selection | – | – | – | 0 of 94 (0%) |
| 1st round of selection | $4 \times 10^{13}$ | $2 \times 10^{7}$ | $5 \times 10^{-7}$ | 47 of 94 (50%) |
| 2nd round of selection | $2 \times 10^{12}$ | $4 \times 10^{9}$ | $2 \times 10^{-3}$ | 75 of 94 (80%) |
| **B** | | | | |
| Before selection | – | – | – | n.d. |
| 1st round of selection | $2 \times 10^{13}$ | $2 \times 10^{6}$ | $1 \times 10^{-7}$ | n.d. |
| 2nd round of selection | $4 \times 10^{13}$ | $1 \times 10^{7}$ | $2.5 \times 10^{-7}$ | 0 of 94 (0%) |
| 3rd round of selection | $6 \times 10^{13}$ | $5 \times 10^{8}$ | $8.3 \times 10^{-6}$ | 7 of 93 (8%) |

[a]Clones have been stated as positive if $OD_{490\ nm}$ was three times higher than background; cfu, colony forming unit; n.d., not determined.

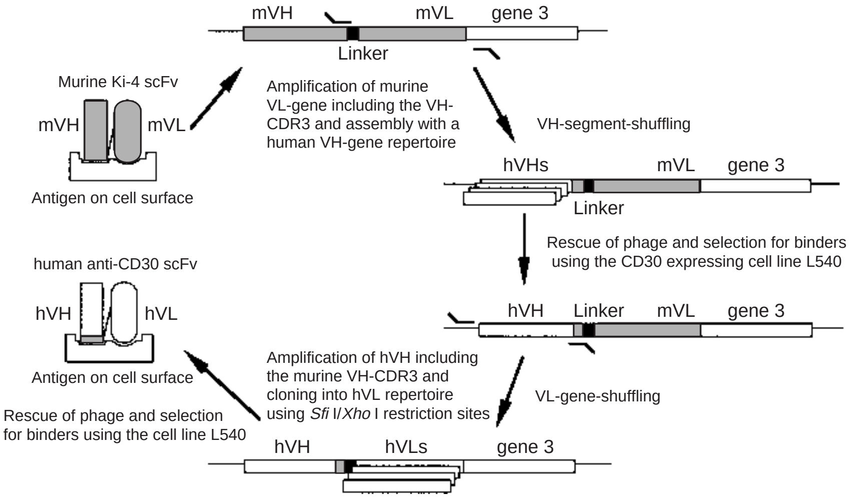

**Figure 1** Schematic drawing of the chain-shuffling procedure used for the guided selection of the human anti-CD30 scFv hAK30. The gene 3 encodes the phage minor coat protein p3 and is part of the phagmid vector pCANTAB6.

different anti-CD30 hybridomas or approx. 0.5 μg ml$^{-1}$ purified Ki-4 Fab-fragment, mKi-4 scFv, A12 scFv or hAK30 scFv in 1 ml 10% FCS medium, respectively, to ensure an excess of antibody against the CD30 receptor on the cell surfaces. After 2 h, the cells were washed three times in 10 ml 10% FCS medium by centrifugation (300 ***g***) and resuspension to remove unbound antibodies, before the cells were incubated for further 24 h. 100 μl of cell-free supernatants were checked for the level of shed extracellular CD30 receptor using the CD30 (Ki-1 antigen)-ELISA kit (DAKO, Glostrup, Denmark). Relative $OD_{450}$ extinction was determined and compared to the sCD30-level of cells incubated with hybridoma supernatant of an anti-GD2 antibody (BW702) as control.

### RESULTS

### Cloning of V genes and selection of the half-human and human anti-CD30 scFv

To retrieve a fully human anti-CD30 scFv, the strategy of ‘guided selection’ (Figure 1) was followed using a recently-cloned murine anti-CD30 scFv (mKi-4 scFv) as guiding molecule. First, the CDR3-linker-VL gene fragment of the murine anti-CD30 Ki-4 scFv was combined with a repertoire of CDR3-truncated human VH genes taken from a repertoire of $1.8 \times 10^{8}$ human scFv clones (Marks et al, 1991). Phage displaying these combinatorial scFvs were selected for binding to the CD30-positive cell line L540. As documented in Table 1A, two rounds of selection and amplification were sufficient to enrich for CD30-binding, half-human scFv bearing phage (human VH-murine VL) up to 80%, as determined in a whole-cell ELISA using L540 cells. DNA-fingerprint analysis of 12 individual clones with the restriction enzyme BstN I revealed five different patterns in DNA-gel electrophoresis (Figure 2A). The scFv-genes of five representative clones were sequenced and the deduced amino-acid sequences were compared with the Kabat database and the Sanger Centre database of human VH-genes to determine their V-gene family and their closest germline match.

As depicted in Table 2, all five V-genes belong to the VH-I family, with two VH-genes showing the highest homology to the VH DP-75 segment. This segment is also the gene with the highest homology towards the murine VH sequence. The deduced amino-acid sequences of the human and murine Ki-4 heavy-chain CDR1 and CDR2 show a homology of 23–50% (Table 3). However, structural analyses, as far as they can be predicted from the amino-acid sequence (Chothia et al, 1989; 1992), revealed that similar classes of canonical structures for the human and the murine VH-genes occurred.

*© 2000 Cancer Research Campaign*

**Table 2** Deduced amino acid sequences of selected VH- and VL-genes

Heavy chains:

| | FR1 | CDR1 | FR2 | CDR2 | FR3 | CDR3 | FR4 |
|---|---|---|---|---|---|---|---|
| Numbering | 1 10 20 30 | | 40 50 | 60 | 70 80 90 | 100 | 110 |
| mVH Ki–4 | QVKLQESGTELAKPGAAVKMSCKASGYTFT | **DYWMH** | WVKQRPGQGLEWIG | **YINPNTAYTDYNQKFKD** | KATLTADKSSSTAYMQLRSLTSEDSAVYYCAK | **KTTQTTWGFPF** | WGQGTTVTVSS |
| hVH A9 | --Q-----A-VK----S--L---------- | **NHFI-** | --R-A-------M- | **T---SSGS-T-A-R-QG** | RV-M-R-T-TR-V--E-SR--HD-T------- | **-----------** | ----------- |
| hVH A3 | --Q--Q--A-VK----S--V---------- | **S-GIN** | -LR-A-------M- | **G-I-IFGTAN-T-R-Q-** | RL-I---D-T--S--E-SD-----T------- | **-----------** | ----------- |
| hVH A4 | --Q-VQ--A-VK---SS--V------G--S | **SSSIS** | --R-A-------M- | **G-I-SFGTAN-A-R-QG** | RV-I---E-T-----E-S--R---T-----T- | **-----------** | ----------- |
| hVH A2 | --Q-VQ--A-VK--R-S--V---T------ | **G-Y--** | --R-A----F--M- | **W-D--GGA-T-A---QG** | RL---R-T-IN----D-SR---D-T------- | **-----------** | ----------- |
| hVH A12(hAK30) | --Q--Q--A-VK---SS--V---T------ | **G-Y--** | --R-A----F--M- | **W-D--SGA-T-A---QG** | RLI-SR-T-IN----E--R---D-T------- | **-----------** | ----------- |
| | | [H1] | | [H2] | | | |

Light chains:

| | FR1 | CDR1 | FR2 | CDR2 | FR3 | CDR3 | FR4 |
|---|---|---|---|---|---|---|---|
| Numbering | 1 10 20 | 30 | 40 | 50 | 60 70 80 | 90 | 100 |
| mVL Ki–4 | DIVLTQSPKSMAMSVGERVTLSC | **KASENVDSFVS** | WYQQKPGQSPKLLIY | **GASNRYT** | GVPDRFAGSGSGRDFTLTISSVQAEDLADYHC | **GQNYRYPLT** | FGAGTKLEIK |
| hVL hAK30hVH | ---M----STLSA---D---IT- | **R--QG-YQWLA** | -------KA-N---- | **K--HL-N** | ---S--S-----T--------L-PD-F-T-Y- | **Q-LNS----** | --G---V--- |
| | | [L1] | | [L2] | [L3 | ] | |

Amino acid sequences of selected VH and VL fragments are aligned towards parental murine Ki-4 fragments. Different amino acids are indicated. Numbering is according to Kabat and Wu (1997). Definition of CDR-loops (H1, H2, L1, L2, L3) is according to Chothia et al (1989; 1992); FR,. framework region; CDR, complementarity determining region.

*© 2000 Cancer Research Campaign*

**Table 3** V-gene classifications, structural predictions of the CDR-loops and homologies of V-genes involved in the chain-shuffling procedure

| V-genes | V-gene family[a] | Predicted canonical structure of CDR loops[b] H/L 1 | H/L 2 | H/L 3 | Human germline gene with closest deduced protein sequence[c] | Amino-acid sequence homology (%) of CDRs towards murine Ki-4 |
|---|---|---|---|---|---|---|
| mVH-Ki-4 | Mo-VH VII | 1 | 2 | n.a. | VH DP-75 | 100 |
| hVH A9 | Hu-VH1 | 1 | 2/3 | n.a. | VH hv1f10t | 36 |
| hVH A3 | Hu-VH1 | 1 | 2 | n.a. | VH VHGL-1.8 | 32 |
| hVH A4 | Hu-VH1 | 1 | 2 | n.a. | VH DP-10 | 23 |
| hVH E2 | Hu-VH1 | 1 | 2/3 | n.a. | VH DP-75 | 50 |
| hVH A12 (hAK30) | Hu-VH1 | 1 | 2/3 | n.a. | VH DP-75 | 50 |
| mVL-Ki-4 | Mo-VκXXI | 2 | 1 | 1 | Vκ DPK-24 | 100 |
| hVL-4 (hAK30) | Hu-Vκ1 | 2 | 1 | 1 | VκL12a+ | 41 |

[a]V-gene families assigned to Kabat database (http://immuno.bme.nwu.edu/famgroup.html); [b]Canonical structures were determined according to Chothia et al (1989; 1992); [c]germline genes assigned to Sanger Centre database (http://www.sanger.ac.uk); n.a., not applicable

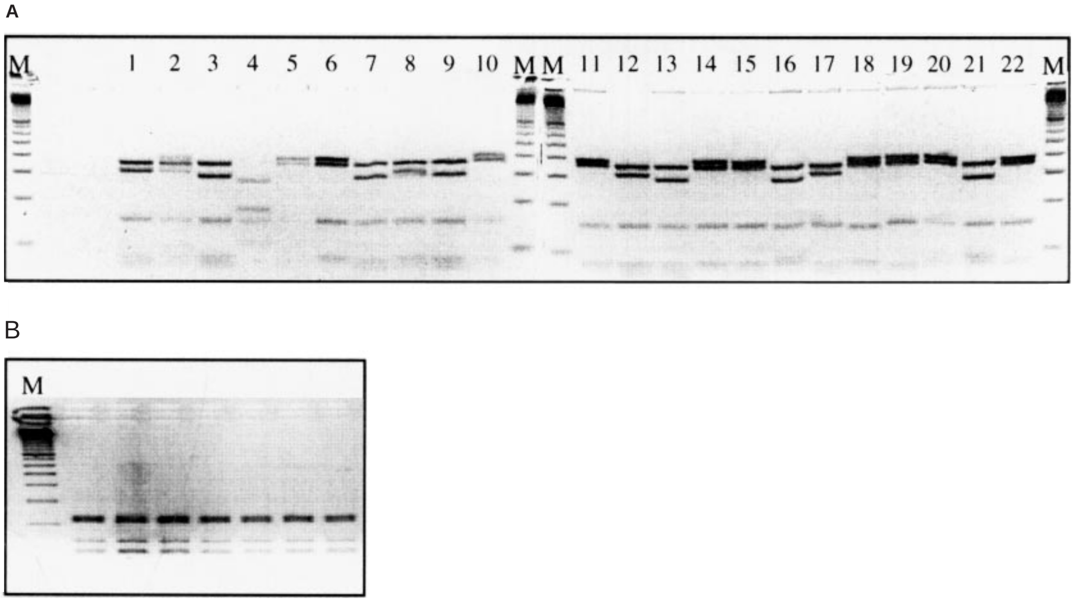

**Figure 2** Bst NI fingerprint-analysis of positive scFv clones determined by whole-cell ELISA using cell line L540. The scFv inserts were PCR-amplified from individual colonies using vector-based primers according to Marks et al (1991). The products were digested with Bst NI and analysed on agarose gels. M, 100 bp molecular weight marker. (**A**) Digests from colonies with half-human scFvs after 1 round of selection (lanes 1 to 10) and 2 rounds of selection (lanes 11 to 22). (**B**) Lanes 1 to 7 are digest, from colonies with human scFvs after 3 rounds of selection.

The five selected human VH-genes were PCR-amplified and cloned into the phagemid vector pCANTAB6. After sequencing the VH-genes once more, they were pooled and cloned into a $4.5 \times 10^6$ member human (h)VL phage antibody library in pHEN-1 (Marks et al, 1991) resulting in a combinatorial library of $8 \times 10^6$ individual clones. Three rounds of phage selection and amplification were performed using the Hodgkin-derived cell line L540, which resulted in 8% binders in a whole-cell ELISA (Table 1B). DNA-fingerprint analysis showed that all positive clones contain the same human VH- (from half-human clone A12) and human VL- gene (Figure 2B), which was subsequently confirmed by sequencing two of these clones. The deduced amino-acid sequence of the selected human VL-gene (Table 2) shows that it possesses a 41%-homology to the parental murine VL-gene in the CDR regions and retains similar structural elements (Table 3). The DNA-sequence of the final human anti-CD30 scFv hAK30 was submitted to GenBank (accession number AF117956).

### Binding properties of the half-human and human anti-CD30 scFvs

To verify binding specificity of the scFvs against the CD30-epitope recognized by the monoclonal Ki-4 antibody, competition experiments were performed and evaluated by FACS analysis. As shown in Figure 3, binding of the selected scFvs displayed on phage was partially but specifically blocked by the parental Ki-4 moab, but not by the monoclonal Ki-3 antibody, which recognizes a different epitope on the CD30 antigen (shown for mKi-4 scFv, h/mA12 scFv, and human hAK30 scFv). The scF-v-genes were subsequently expressed in *E. coli* non-suppressor strain HB2151

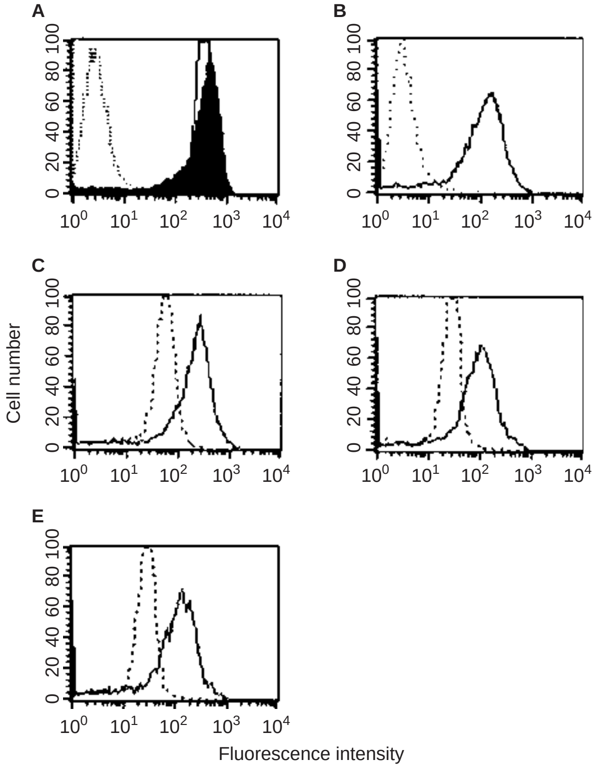

**Figure 3** Histograms of FACS-analysis for determination of the CD30-epitope specificity of the selected anti-CD30 scFvs. (**A**) L540-cells were incubated with anti-CD30 Ki-3- (filled area), Ki-4 (unfilled area) hybridoma-supernatant or PBS (dotted line) and binding was detected by FITC-conjugated goat-anti-mouse IgG antibody. (**B**) L540 cells were incubated with phage displaying no scFv (dotted line) or phage displaying half-human scFv A12 (black line). L540 cells were incubated with phage-antibodies mKi-4 (**C**), h/mA12 (**D**) or hAK30 (**E**) and additionally with anti-CD30 moab Ki-3- (black line) or Ki-4- (dotted line) hybridoma-supernatant, respectively. Binding of phage-antibodies was subsequently detected using sheep-anti-M13-serum and FITC-conjugated rabbit-anti-sheep-IgG antibody.

**Table 4** Apparent affinities of anti-CD30 antibodies

| Anti-CD30 antibody | $K_d$ (M) |
|---|---|
| moab Ki-4 | $4 \times 10^{-10}$ |
| Ki-4 Fab-fragment | $5 \times 10^{-9}$ |
| mKi-4 scFv | $3 \times 10^{-9}$ |
| h/mA12 scFv | $7 \times 10^{-9}$ |
| hAK30 scFv | $3 \times 10^{-8}$ |

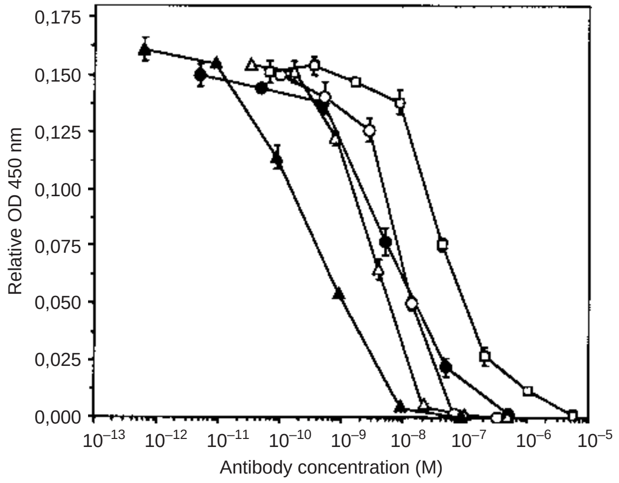

**Figure 4** Apparent binding affinities of anti-CD30 antibodies using recombinant sCD30-His antigen. Dilution series of anti-CD30 antibodies moab Ki-4 —▲—, Ki-4 Fab-fragment —●—, mKi-4 scFv —△—, h/mA12 scFv —○— and hAK30 scFv —□—were incubated with recombinant sCD30-His protein and unbound antigen was detected by CD30-ELISA (DAKO).

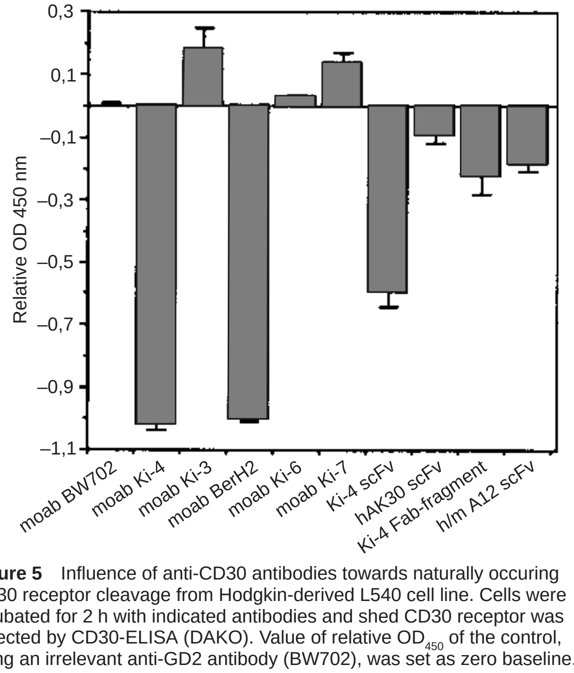

**Figure 5** Influence of anti-CD30 antibodies towards naturally occuring CD30 receptor cleavage from Hodgkin-derived L540 cell line. Cells were incubated for 2 h with indicated antibodies and shed CD30 receptor was detected by CD30-ELISA (DAKO). Value of relative $OD_{450}$ of the control, using an irrelevant anti-GD2 antibody (BW702), was set as zero baseline.

and purified by IMAC. The typical yield of purified scFv was approximately 150 µg $l^{-1}$ bacterial culture performing a standard periplasmic extraction, or twice as much using a modified protocol (see Material and methods).

The relative binding affinities were determined by ELISA using a defined concentration of purified sCD30-His protein as antigen and dilution series of the indicated anti-CD30 antibodies (Figure 4). Purified scFv antibodies consisted of at least 95% monomeric molecules demonstrated by gelfiltration (data not shown). Antigen and antibodies were incubated in solution and unbound antigen was subsequently quantified with a coated anti-CD30 antibody, recognizing the same epitope as the investigated antibodies. The antibody concentration at which 50% of the antigen was bound at equilibrium was taken as the apparent $K_d$. The relative affinities of the moab Ki-4 Fab-fragment, the murine Ki-4 scFv and the half-human anti-CD30 scFv A12 are approximately 10-fold higher than the affinity of the human scFv hAK30 (Table 4), but 10-fold lower than the whole, bivalent monoclonal antibody Ki-4.

### Shedding-inhibition of the extracellular part of the CD30 receptor

To investigate the influence of our recombinant anti-CD30 antibodies on the shedding of the extracellular part of the CD30 receptor, L540 cells were incubated with supernatants of different anti-CD30 hybridomas or purified recombinant anti-CD30 scFvs, respectively. After 2 h, the cells were thoroughly washed with

*© 2000 Cancer Research Campaign*

medium to deplete unbound antibodies. After another 24 h of incubation, supernatants were checked for sCD30-level in a CD30 (Ki-1 Antigen) ELISA kit (DAKO) in duplicates. Figure 5 shows that, as was described by Horn-Lohrens et al (1995), Ki-4 and BerH2 strongly inhibit the shedding of the extracellular part of CD30 receptor (sCD30), whereas Ki-3, Ki-6 and Ki-7 increased the sCD30 level to different extents. The recombinant Ki-4-derived anti-CD30 antibodies and the monovalent moab Ki-4 Fab fragment exhibit a comparable inhibition of the cleavage of CD30 receptor from L540 cells, although they are not as potent as the bivalent moabs Ki-4 and BerH2.

### DISCUSSION

In this paper, we report the cloning of a human anti-CD30 scFv by guided selection from the murine anti-CD30 scFv Ki-4 using the phage display technology. In contrast to other described procedures (Jespers et al, 1994; Figini et al, 1994), we retained the VH-CDR3 region of the parental murine scFv in this guided selection. This particular region is not only known for its significant importance in determining the binding specificity of an antibody, it is also a highly variable region in every antibody, which makes it less likely to be a major immunogenic part of the molecule. We believe that this region was important in retaining the CD30-epitope specificity of the parental antibody in the human scFv. Watzka et al (1998) describe the humanization through chain-shuffling of an anti-human interferon γ receptor 1 antibody without retaining the VH-CDR3 region of the murine antibody. The resulting fully-human Fab-antibody was antigen specific, but differed in epitope specificity from the parental hybridoma, thereby underlining the importance of the VH-CDR3. This importance of the VH-CDR3 for epitope specificity has also recently been reported by Beiboer et al (2000), thereby confirming our findings.

Selection of phage libraries in this study was performed on a Hodgkin-derived cell line which is known for its high surface expression of CD30 receptor ($10^6$ receptor molecules per cell), rather than by panning on recombinant CD30 antigen. CD30 is part of the TNF receptor family and many ligands and receptors are known to trimerize. It may therefore not be straightforward to retain natural epitopes on recombinant versions of such cell-surface molecules. Therefore, cell panning on CD30-positive cells is a good and valid alternative. Indeed, using recombinant CD30 protein for panning of several human scFv phage repertoires, to date other groups were unsuccessful in retrieving functional anti-CD30 antibodies.

The selection on cells and therefore native CD30 receptor resulted in five different human VH genes with homology in the CDR1 and CDR2 regions between 23–50%, compared with the parental murine Ki-4 heavy chain. This is similar to what was found in the group of Watzka et al (1998) for their selected anti-human interferon γ receptor antibody (45%), and higher than described in the study of Figini et al (1998), in which an anti-ovarian carcinoma Fab fragment was humanized by guided selection. However, more important than sequence homology might be the length of the CDR regions and the canonical structure of the CDR-loops defined by Chothia et al (1989; 1992). This was striking in the case described by Figini et al (1994) retrieving a human anti-phOx Fab fragment by guided selection which shared these structural elements with the parental mouse antibody. In our study, the selected human VH gene with the highest sequence homology towards mKi-4 VH, but probably slightly different canonical folds, is the one of the half-human anti-CD30 scFv A12. This clone was also predominant after the selection (Figure 2A). Additionally, this human VH gene was selected out of five others after shuffling with the human VL-repertoire. The human VL gene of the finally selected human anti-CD30 scFv has the same length and predicted canonical structure of the CDR-regions as the mKi-4 VL gene, and a 41% homology of the deduced amino-acid sequences concerning these regions. This follows the prediction made by Jespers et al (1994) that there may be a strong preference for retaining V-gene segments with identical canonical folds in guided selection procedures.

Expression of the human anti-CD30 scFv hAK30 as soluble fragment revealed a 10-fold lower apparent $K_d$ for the hAK30 scFv compared with the mKi-4 scFv (Table 4). A loss in affinity after a guided selection procedure has also been reported by other groups (Figini et al, 1998). However, the hAK30 scFv reveals an affinity in the nanomolar range and is therefore expected to be adequate for use as targeting moiety in recombinant immunotherapeutics, in particular when re-formatted as bivalent molecule (Tai et al, 1995). The relative affinity of the monovalent Ki-4 Fab-fragment ($5 \times 10^{-9}$ M) is comparable to the value measured for the mKi-4 scFv and underlines the successful cloning of the functional V-genes from the hybridoma Ki-4. The higher affinity of the bivalent moab Ki-4 ($3.7 \times 10^{-10}$ M) most probably is caused by an avidity effect in the assay.

The monoclonal antibody Ki-4, as well as the moab BerH2, significantly inhibit the naturally occurring shedding of the extracellular part of the CD30 receptor, as demonstrated by Horn-Lohrens et al (1995). Since this is a desired property for an anti-CD30 antibody as part of an immunotherapeutic agent, we were especially interested in retaining the epitope-specificity of the moab Ki-4 in our human anti-CD30 antibody. As shown in Figure 3, the epitope-specificity was retained for the murine and the human scFv. Although the binding of phage antibodies was not completely inhibited by moab Ki-4, which might be due to higher avidity effects of phage (displaying up to five scFv molecules on their surface), competition for binding was not observed by addition of moab Ki-3. The anti-sCD30-shedding property was retained as well, although it was significantly weaker for the monovalent anti-CD30 molecules, which correlates with their apparent binding affinities (Figure 5). A bivalent scFv, like a diabody, may even be as potent as the bivalent moab Ki-4 regarding the CD30-shedding inhibition. Whether the human anti-CD30 scFv hAK30 will be as potent as the murine Ki-4 scFv as part of an anti-CD30 immunotherapeutic agent (Klimka et al, 1999) has to be further analysed, e.g. by fusing it to a human-derived toxin gene (Newton et al, 1996) in order to get a fully human, recombinant immunotoxin.

In summary, we have been able to derive a functional human anti-CD30 scFv (hAK30) from the murine anti-CD30 scFv Ki-4 by guided selection using human V-gene repertoires and phage display technology. The hAK30 scFv retains the epitope specificity of its murine counterpart and inhibits the shedding of the CD30 receptor from the cell surface.

### ACKNOWLEDGEMENTS

The authors thank Mrs E van der Linden, Mrs P Henderikx, Mr M Rousch, Mrs G Schön and Mrs S Drillich for their excellent technical assistance. This work was supported in part by ‘Deutsche

© 2000 Cancer Research Campaign 

Forschungsgemeinschaft, SFB 502' and by 'Boehringer Ingelheim Fond'.

### REFERENCES

Barth S, Huhn M, Matthey B, Klimka A, Galinski EA and Engert A (2000) Compatible solute-supported periplasmic expression of functional recombinant proteins under stress conditions. *Appl Environ Microbiol* **66**: 1572–1579

Beiboer SH, Reurs A, Roovers RC, Arends JW, Whitelegg NR, Rees AR and Hoogenboom HR (2000) Guided selection of a pan carcinoma specific antibody reveals similar binding characteristics yet structural divergence between the original murine antibody and its human equivalent. *J Mol Biol* **296**: 833–849

Bosslet K, Mennel HD, Rodden F, Bauer BL, Wagner F, Altmannsberger A, Sedlacek HH. and Wiegandt H (1989) Monoclonal antibodies against epitopes on ganglioside GD2 and its lactones. Markers for gliomas and neuroblastomas. *Cancer Immunol Immunother* **29**: 171–178

Brüggemann M and Neuberger MS (1996) Strategies for expressing human antibody repertoires in transgenic mice. *Immunol Today* **17**: 391–397

Chothia C, Lesk AM, Tramontano A, Levitt M, Smith-Gill SJ, Air G, Sheriff S, Padlan EA, Davies D, Tulip WR and et al (1989) Conformations of immunoglobulin hypervariable regions. *Nature* **342**: 877–883

Chothia C, Lesk AM, Gherardi E, Tomlinson IM, Walter G, Marks JD, Llewelyn MB and Winter G (1992) Structural repertoire of the human VH segments. *Mol Biol* **227**: 799–817

Diehl V, Kirchner HH, Schaadt M, Fonatsch C, Stein H, Gerdes J and Boie C (1981) Hodgkin's disease: establishment and characterization of four in vitro cell lines. *J Cancer Res Clin Oncol* **101**: 111–124

Dower WJ, Miller JF and Ragsdale CW (1988) High efficiency transformation of *E. coli* by high voltage electroporation. *Nucleic Acids Res* **16**: 6127–6145

Figini M, Marks JD, Winter G and Griffiths AD (1994) In vitro assembly of repertoires of antibody chains on the surface of phage by renaturation. *J Mol Biol* **239**: 68–78

Figini M, Obici L, Mezzanzanica D, Griffiths A, Colnaghi MI, Winter G and Canevari S (1998) Panning phage antibody libraries on cells: isolation of human Fab fragments against ovarian carcinoma using guided selection. *Cancer Res* **58**: 991–996

Gruss HJ and Dower SK (1995) The TNF ligand superfamily and its relevance for human diseases. *Cytokines Mol Ther* **1**: 75–105

Hoogenboom HR (1997) Designing and optimizing library selection strategies for generating high-affinity antibodies. *Trends Biotechnol* **15**: 62–70

Hoogenboom HR, de Bruine AP, Hufton SE, Hoet RM, Arends JW and Roovers RC (1998) Antibody phage display technology and its applications. *Immunotechnology* **4**: 1–20

Hoogenboom HR, Lutgerink JT, Pelsers MM, Rousch MJ, Coote J, Van Neer N, DeBruine A, Van Nieuwenhoven FA, Glatz JF and Arends JW (1999) Selection-dominant and nonaccessible epitopes on cell-surface receptors revealed by cell-panning with a large phage antibody library. *Eur J Biochem* **260**: 274–284

Horn-Lohrens O, Tiemann M, Lange H, Kobarg J, Hafner M, Hansen H, Sterry W, Parwaresch RM and Lemke H (1995) Shedding of the soluble form of CD30 from the Hodgkin-analogous cell line L540 is strongly inhibited by a new CD30-specific antibody (Ki-4). *Int J Cancer* **60**: 539–544

Huston JS, McCartney J, Tai MS, Mottola-Hartshorn C, Jin D, Warren F, Keck P and Oppermann H (1993) Medical applications of single-chain antibodies. *Int Rev Immunol* **10**: 195–217

Jespers LS, Roberts A, Mahler SM, Winter G and Hoogenboom HR (1994) Guiding the selection of human antibodies from phage display repertoires to a single epitope of an antigen. *Bio/Technology* **12**: 899–903

Kabat EA and Wu TT (1991) Identical V region amino acid sequences and segments of sequences in antibodies of different specificities. Relative contributions of VH and VL genes, minigenes, and complementarity-determining regions to binding of antibody-combining sites. *J Immunol* **147**: 1709–1719

Khazaeli MB, Conry RM and LoBuglio AF (1994) Human immune response to monoclonal antibodies. *J Immunother* **15**: 42–52

Klimka A, Barth S, Matthey B, Roovers R, Lemke H, Hansen H, Arends JW, Diehl V, Hoogenboom HR and Engert A (1999) An anti-CD30 single-chain Fv selected by phage display and fused to *Pseudomonas* exotoxin A (Ki-(scFv)-ETA) is a potent immunotoxin against a Hodgkin-derived cell line. *Br J Cancer* **80**: 1214–1222

Köhler G and Milstein C (1975) Continuous cultures of fused cells secreting antibody of predefined specificity. *Nature* **256**: 495–497

Marks JD, Hoogenboom HR, Bonnert TP, McCafferty J, Griffiths AD and Winter G (1991) By-passing immunization. Human antibodies from V-gene libraries displayed on phage. *J Mol Biol* **222**: 581–597

McCafferty J, Fitzgerald KJ, Earnshaw J, Chiswell DJ, Link J, Smith R and Kenten J (1994) Selection and rapid purification of murine antibody fragments that bind a transition-state analog by phage display. *Appl Biochem Biotechnol* **47**: 157–171

McCall AM, Amoroso AR, Sautes C, Marks JD and Weiner LM (1998) Characterization of anti-mouse Fc gamma RII single-chain Fv fragments derived from human phage display libraries. *Immunotechnology* **4**: 71–87

Meredith RF, Khazaeli MB, Grizzle WE, Orr RA, Plott G, Urist MM, Liu T, Russell CD, Wheeler RH, Schlom J and et al (1993) Direct localization comparison of murine and chimeric B72.3 antibodies in patients with colon cancer. *Hum Antibodies Hybridomas* **4**: 190–197

Newton DL, Xue Y, Olson KA, Fett JW and Rybak SM (1996) Angiogenin single-chain immunofusions: influence of peptide linkers and spacers between fusion protein domains. *Biochemistry* **35**: 545–553

Persic L, Horn IR, Rybak S, Cattaneo A, Hoogenboom HR and Bradbury A (1999) Single-chain variable fragments selected on the 57–76 p21Ras neutralising epitope from phage antibody libraries recognise the parental protein. *FEBS Lett* **443**: 112–116

Sanger F, Nicklen S and Coulson AR (1977) DNA sequencing with chain-terminating inhibitors. *Proc Natl Acad Sci USA* **74**: 5463–5467

Schnell R, Linnartz C, Katouzi AA, Schon G, Bohlen H, Horn-Lohrens O, Parwaresch RM, Lange H, Diehl V, Lemke H and et al (1995) Development of new ricin A-chain immunotoxins with potent anti-tumor effects against human Hodgkin cells in vitro and disseminated Hodgkin tumors in SCID mice using high-affinity monoclonal antibodies directed against the CD30 antigen. *Int J Cancer* **63**: 238–244

Schwab U, Stein H, Gerdes J, Lemke H, Kirchner H, Schaadt M and Diehl V (1982) Production of a monoclonal antibody specific for Hodgkin and Sternberg–Reed cells of Hodgkin's disease and a subset of normal lymphoid cells. *Nature* **299**: 65–67

Schwarting R, Gerdes J, Durkop H, Falini B, Pileri S and Stein H (1989) BER-H2: a new anti-Ki-1 (CD30) monoclonal antibody directed at a formol-resistant epitope. *Blood* **74**: 1678–1689

Shawler DL, Bartholomew RM, Smith LM and Dillman RO (1985) Human immune response to multiple injections of murine monoclonal IgG. *J Immunol* **135**: 1530–1535

Smith TJ (1993) Purification of mouse antibodies and Fab fragments. *Methods Cell Biol* **37**: 75–93

Tai MS, McCartney JE, Adams GP, Jin D, Hudziak RM, Oppermann H, Laminet AA, Bookman MA, Wolf EJ and Liu S (1995) Targeting c-erbB-2 expressing tumors using single-chain Fv monomers and dimers. *Cancer Res* **55 (23 Suppl)**: 5983s–5989s

Watzka H, Pfizenmaier K and Moosmayer D (1998) Guided selection of antibody fragments specific for human interferon gamma receptor 1 from a human VH- and VL-gene repertoire. *Immunotechnology* **3**: 279–291

Winter G, Griffiths AD, Hawkins RE and Hoogenboom HR (1994) Making antibodies by phage display technology. *Annu Rev Immunol* **12**: 433–455

 *© 2000 Cancer Research Campaign*
